# Overexpression of hTERT increases stem-like properties and decreases spontaneous differentiation in human mesenchymal stem cell lines

**DOI:** 10.1186/1423-0127-17-64

**Published:** 2010-07-29

**Authors:** Chih-Chien Tsai, Chun-Li Chen, Hwa-Chung Liu, Yi-Ting Lee, Hsei-Wei Wang, Lein-Tuan Hou, Shih-Chieh Hung

**Affiliations:** 1 Stem Cell Laboratory, Department of Medical Research & Education and Orthopaedics & Traumatology, Veterans General Hospital, Taipei, Taiwan; 2 Graduate Institute of Dental Sciences and Department of Periodontology, National Taiwan University, Taipei, Taiwan; 3 Graduate Institute of Medical Engineering & Department of Orthopedics, College of Medicine, National Taiwan University, Taipei, Taiwan; 4 Institute of Clinical Medicine & Department and Institute of Pharmacology, School of Medicine, National Yang-Ming University, Taipei, Taiwan; 5 Institute of Microbiology and Immunology, National Yang-Ming University, Taipei, Taiwan

## Abstract

To overcome loss of stem-like properties and spontaneous differentiation those hinder the expansion and application of human mesenchymal stem cells (hMSCs), we have clonally isolated permanent and stable human MSC lines by ectopic overexpression of primary cell cultures of hMSCs with HPV 16 E6E7 and human telomerase reverse transcriptase (hTERT) genes. These cells were found to have a differentiation potential far beyond the ordinary hMSCs. They expressed trophoectoderm and germline specific markers upon differentiation with BMP4 and retinoic acid, respectively. Furthermore, they displayed higher osteogenic and neural differentiation efficiency than primary hMSCs or hMSCs expressed HPV16 E6E7 alone with a decrease in methylation level as proven by a global CpG island methylation profile analysis. Notably, the demethylated CpG islands were highly associated with development and differentiation associated genes. Principal component analysis further pointed out the expression profile of the cells converged toward embryonic stem cells. These data demonstrate these cells not only are a useful tool for the studies of cell differentiation both for the mesenchymal and neurogenic lineages, but also provide a valuable source of cells for cell therapy studies in animal models of skeletal and neurological disorders.

## Introduction

Bone marrow derived human mesenchymal stem cells (hMSCs) are considered one of the most promising and prospective resources for cell and gene therapy in mesenchymal and non-mesenchymal applications because of their great self-renewal and versatile plasticity in vitro and in vivo [1]. However, there are still two major hindrances, loss of stem-like properties, namely self-renewal and multipotency, and spontaneous differentiation, encountered during in vitro expansion of MSCs [2]. Loss of stem-like properties could be defined as diminished replication, altered functionality [3], and deteriorated potential for differentiation [4]. Spontaneous differentiation, known as the emergence of lineage-specific markers without any directed differentiation, would diminish the proportion of undifferentiated stem cells, and therefore compromised the benefit of hMSCs for clinical application. Thus, identifying methods for inhibiting loss of stem-like properties and spontaneous differentiation, and reversing hMSCs to a more primitive state has attracted great research interest.

In a previous attempt to immortalize hMSCs with increased life span, we have established a cell line-KP by transferring HPV16 E6E7 genes into hMSCs [5]. Though KP successfully overcomes the drawback of cellular senescence and could be passaged over 100 population doublings (PDs), the phenomenon of spontaneous differentiation could not be avoided [6]. Telomerase, known to maintain the telomere length, has been indicated to play a role in self-renewal and pluripotency of embryonic stem cells (ESCs) [7]. However, hMSCs express no telomerase activity with telomere shortening in a rate similar to non-stem cells (30-120 bp/population doubling), and cease to divide when the telomere length is less than 10 kb [8]. Besides, ectopic expression of human telomerase reverse transcriptase (hTERT), the catalytic component of telomerase, has been proven not only to bypass cellular senescence and extend life span [9], but also to influence differentiation potential [10]. Notably, a recent report has unraveled a fascinating fact that TERT might play a crucial role in gene regulation directly or indirectly, which finally caused profound changes in gene expressions of mouse skin [11]. What's most important, the authors further demonstrated that the effect of TERT on gene regulation is irrelevant to its catalytic enzyme action at telomere ends [11].

In mammals, DNA methylation of cytosines in cytosine guanine dinucleotide (CpG) islands, known to mediate epigenetic gene silencing [12,13], plays pivotal roles in embryonic development [14-16] and ESC differentiation [17]. For example, treating ESCs or somatic cells with demethylation agent such as 5-azacytidine (5-AzaC) resulted in dedifferentiation, thereby pointing out the association of DNA methylation with the differentiation state [18-20]. These results also imply methods that reverse the differentiation state of stem or progenitor cells will induce changes in DNA methylation patterns [17].

In this study, we hypothesized, after ectopic expression of HPV16 E6E7 and hTERT, hMSCs would bypass loss of stem-like properties and block spontaneous differentiation with changes in DNA methylation patterns. Meanwhile, we also tried to demonstrate the heightened differentiation potential of HPV16 E6E7 and hTERT-transfected hMSCs by directing germline and trophoectoderm differentiation. Finally, the roles of DNA methylation-modification factors, such as DNA methyltransferases (DNMTs) in the reversion of hMSCs to a more primitive state would be explored.

## Materials and methods

### Cell Cultures

Primary hMSCs were obtained from the Tulane Center for Preparation and Distribution of Adult Stem Cells (http://www.som.tulane.edu/gene_therapy/). The cells were grown in alpha minimal essential medium (αMEM; GIBCO/BRL, Carlsbad, CA; http://www.invitrogen.com) supplemented with 16.6% fetal bovine serum (FBS), 100 U/ml penicillin, 100 μg/ml streptomycin, and 2 mM L-glutamine (GIBCO/BRL) at 37°C under 5% CO2 atmosphere. The medium was changed twice per week and a subculture was performed after they reached about 80% confluency.

The hMSC strain (KP) was developed by transfection with the type 16 human papilloma virus proteins E6E7 as described previously [6]. This strain is grown in DMEM-LG (GIBCO/BRL) supplemented with 10% FBS, 100 U/ml penicillin, 100 μg/ml streptomycin, and 2 mM L-glutamine. The medium was changed twice per week and a subculture was performed at 1:3 to 1:5 split every week. Using flow cytometry, cells express CD29, CD44, CD90, CD105, SH2, and SH3.

### DNA Delivery Methods

KP cells were transfected with phTERT-IRES2-EGFP, which was generated by inserting a 3.45-kb *EcoRI-EcoRI *fragment containing the hTERT cDNA into pIRSE2-EGFP (Clontech, Palo Alto, CA, http://www.clontech.com) using Nucleofector technology as recommended by the manufacturer (Amaxa Biosystems, Cologne, Germany, http://www.amaxa.com). The efficiency of transfection as evaluated by the expression of EGFP was around 70%. The cells were then suspended in an appropriate volume of 20% FBS-supplemented DMEM-LG medium, seeded in 96 well plate for selecting single cell clone by neomycin (400 μg/ml).

### Reverse Transcription-Polymerase Chain Reaction (RT-PCR)

Total RNA was extracted using the Tri Reagent (Sigma, St. Louis, MO. http://www.sigmaaldrich.com) according to the manufacturer's specifications. First strand cDNA synthesis was performed using Superscript III reverse transcriptase (Invitrogen, Carlsbad, CA, http://www.invitrogen.com), Random primer (Invitrogen), 10 mM dNTPs (Invitrogen), 5× First Strand synthesis buffer, 0.1 M DTT, and RNaseOUT ribonuclease RNase inhibitor (Invitrogen). PCR was performed using cDNA as the template in a 50 μl reaction mixture containing a specific primer pair of each cDNA according to the published sequences. The reaction products were resolved by electrophoresis on a 1.5% agarose gel and visualized with ethidium bromide. Sequences of PCR primers and NCBI reference sequence numbers were listed in Additional file 1.

### Real-Time PCR

Real-Time PCR was performed using an ABI PRISM 7700 sequence detection system (Applied Biosystem, Foster City, CA, http://www.appliedbiosystems.com) and the TaqMan Universal Master Mix (Applied Biosystems). Analysis of the results was carried out using the software supplied with the machine. The software calculates each gene expression relative to the β-actin housekeeper gene (delta CT) and then relative to controls (delta delta CT) using the fluorescence threshold of the amplification reaction and the comparative CT method. Sequences of PCR primers, probe and PCR conditions can be provided on request.

### Differentiation Protocols

Trophoectoderm differentiation protocol was modified from a previous method [21]. Cells at 50% of confluence were treated with 100 ng/mL BMP4 (R&D Systems, Minneapolis, MN, http://www.rndsystems.com) in DMEM-LG supplemented with 10% FBS. Medium was changed twice per week. Germline differentiation protocol was performed with a protocol modified from previous report [22]. In brief, cells were plated at a density of 1~2 × 10^4 ^cells/cm^2 ^in DMEM-LG supplemented with 10% FBS and 2 μM retinoic acid (RA, Sigma) with medium change twice per week. For osteogenic differentiation, cells were seeded at a density of 10^4 ^cells/cm^2 ^and induced in DMEM-LG supplemented with 10% FBS, 50 μg/ml ascorbate-2 phosphate (Nacalai, Kyoto, Japan, http://www.nacalai.co.jp), 10^-8 ^M dexamethasone (Sigma) and 10 mM β-glycerophosphate (Sigma) with medium change twice per week. For neurogenic differentiation [23], 100 ng/ml recombinant human Noggin (R&D Systems) was added into the serum-free DMEM-LG culture medium.

### Histochemical Studies

Cells were fixed in 2% paraformaldehyde for 10 min and stained for alkaline phosphatase activity and in vitro mineralization by Alizarin red-S [5] to reveal osteogenic differentiation. After washing 5 times with PBS, stained cultures were photographed.

### DNA Methylation Array

#### DNA preparation

Genomic DNA was extracted from samples using QIAamp^® ^DNA mini kit (Qiagen GmbH, Hilden, Germany, http://www.qiagen.com) according to the manufacturer's protocol.

#### aPRIMES

1 μg genomic DNA was restricted to completion with 10 U *Mse*I at 37°C in a final volume of 10 μl in the buffer prepared with the 10 × One-Phor-All Buffer *PLUS *(GE Healthcare Bio-science Corp., Piscataway, NJ, http://www.gehealthcare.com). Heat inactivation was carried out at 65°C for 20 min. *Mse*I fragments were then subjected to ligation with PCR linkers, *Mse*I linker-S (5'-TAA CTA GCA TGC-3') and *Mse*I linker-L (5'-AGT GGG ATT CCG CAT GCT AGT-3') overnight. Half of the resulting ligated *Mse*I fragments were digested with the restriction enzyme McrBC (New England Biolabs, Beverly, MA, http://www.neb.com) for 3 h following the conditions recommended by the supplier. The other half of the *Mse*I fragments were digested with the three methylation-sensitive endonucleases *Hpa*II (New England Biolabs; recognition site CCGG, 3 h, 37°C), *Hha*I (New England Biolabs; recognition site CGCG, 3 h, 37°C) and *Bst*UI (New England Biolabs; recognition site CGCG, 3 h, 60°C) according to the recommendations of the supplier. Digested DNA fragments were then treated with 1 μl Proteinase K (Invitrogen) for 1 h at 37°C with subsequent heat inactivation at 80°C for 10 min. For the LM-PCR steps, 2× PCR Master Mix (Promega, Madison, WI, http://www.promega.com) was added to a final volume of 50 μl. A MJ thermocycler was programmed to 68°C for 10 min, followed by 27 cycle loops at 94°C (40 s), 57°C (30 s) and 68°C (75 s). Final elongation was carried out at 72°C for 10 min. PCR products were purified by ethanol precipitation. DNA was eluted in 50 μl nuclease free H_2_O.

#### Labeling and hybridization to microarrays

Both the *Hpa*II/*Hha*I/*Bstu*I-digested and the McrBC-digested samples were differentially labeled with Cy5- or Cy3-conjugated dUTP by use of an Agilent Genomic DNA Labeling Kit PLUS (Agilent Technologies, Palo Alto, CA, http://www.agilent.com). Labeled targets were subsequently cleanup by the use of a Centricon YM-30 column (Millipore, Billerica, MA, http://www.millipore.com), pooled and mixed in a 500-μl hybridization mixtures with 50 μg of human Cot-1 DNA (Invitrogen) in 1× hybridization buffer (Agilent Technologies). Before hybridization to the array, the hybridization mixtures were denatured at 95°C for 3 min and incubated at 37°C for 30 min. To remove any precipitate, the mixture was centrifuged at ≥ 14,000 × *g *for 5 min and the supernatant was transferred to a new tube. The labeled and denatured DNA target was then hybridized to human CpG island microarray (G4492A, Agilent Technologies, USA) at 65°C for 40 h. The arrays were washed with 0.5 × SSC/0.005% Triton X-102 (wash 1) at room temperature for 5 min, and then with 0.1 × SSC/0.005% Triton X-102 (wash 2) at 37°C for 5 min.

#### Image and microarray data analysis

After drying by nitrogen gun blowing, microarrays were scanned with an Agilent microarray scanner (Agilent Technologies) at 535 nm and 625 nm for Cy3 and Cy5, respectively. Scanned images were analyzed by Feature extraction 9.1 software (Agilent Technologies) to quantify signal and background intensity for each feature. Microarray data were firstly normalized with print-tip loess, followed by background-correction, normalization and analysis by the limma package within the R environment (version 2.1.0). The methylation level was determined as the ratio of Cy5/Cy3 in each spot. The raw data from the array experiments is available from the Gene Expression Omnibus (GEO; http://www.ncbi.nlm.nih.gov/geo) under the series accession number GSE (pending number). For Gene Ontology (GO) analysis of the genes decreased in CpG island methylation, we determined the statistically significant GO terms using the hypergeometric probability distribution. For each GO term, a p-value was calculated representing the probability that the number of genes that are annotated at the term could have been found by chance.

#### Microarray expression data sets and principal component analyses (PCA)

The expression profile of hTERT-transfected hMSCs was implemented by using the Affymetrix™ HG U133 Plus 2.0. The microarray data sets of various normal tissues and ESCs were retrieved from public databases. The ESCs used for microarray analysis were H9 clones and all microarray data are available at GEO under the accession no. of GSM249282, GSM124302 and GSM124362. To determine the similarity of the expression profiles between hTERT-transfected hMSCs and various normal human tissues, MSCs, and ESCs, PCA was performed in 31 Affymetrix™ U133 Plus 2.0 array data. using the Partek^® ^Genomics Suite™ software (Partek Incorporated, St. Louis, MO, http://www.partek.com). All microarray datasets in this paper are available at GEO under the accession no. of GSE7234 and GSE9520.

## Results

### Downregulation of Oct4 and Nanog and upregulation of developmental markers and lineage-specific genes during expansion of primary hMSCs

**Figure 1:**
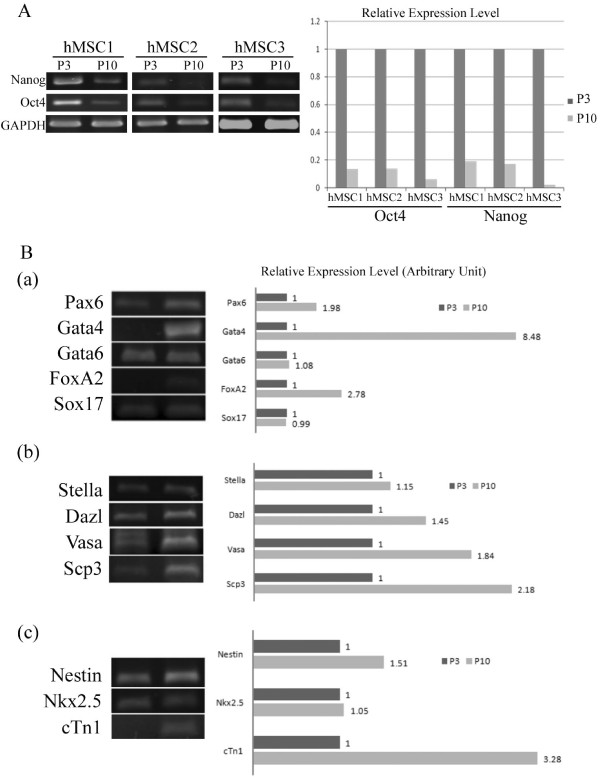
**Differential gene expression between primary cultured passage 3 (P3) and passage 10 (P10)**. **(A) **RT-PCR (left panel) and Real-time RT-PCR (right panel) analysis of pluripotency related genes in MSCs from three individual donors (hMSC-1, -2, -3). **(B) **Differential expression of (a) developmental (b) germline specific (c) lineage specific genes by RT-PCR analysis.

Embryonic transcription factors, such as Oct4 and Nanog, normally expressed in early embryos and ESCs, inhibit tissue-specific genes and enhance self-renewal and pluripotency [24]. To evaluate whether loss of stem-like properties occurred during normal passage of hMSCs, we examined the expression of Oct4 and Nanog in primary hMSCs isolated from three individuals. Semiquantative RT-PCR and real-time RT-PCR analysis revealed higher mRNA levels of Oct4 and Nanog at passage 3 (P3) than at passage 10 (P10) (Figure 1A), suggesting loss of stem-like properties during expansion of primary hMSCs.

ESCs, a powerful tool to study mammalian development, form embryoid bodies (EBs) and express a panel of developmental markers upon removal of feeder layer or leukemia inhibitory factor. To evaluate whether spontaneous differentiation with the expression of developmental markers occurred during normal passage of primary hMSCs, we examined the expression levels of ectoderm (Pax6) [25], primitive endoderm (Gata4 and Gata6) [26] and definitive endoderm (Sox17 and FoxA2) [27] markers by RT-PCR. The expression levels of Pax6, Gata4 and FoxA2 were higher at P10 than at P3 (Figure 1Ba). We next looked at the expression of germline markers [28], and found the expression levels of Stella, Dazl, Vasa and Scp3 were higher at P10 (Figure 1Bb). Finally, we examined two lineage-specific markers expressed in EBs, the neural (Nestin) and cardiac specific genes (Nkx 2.5 and cTn1) and found P10 had higher expression of Nestin and cTn-1 (Figure 1Bc). These results point to upregulation of developmental markers and lineage-specific genes in late-passage primary hMSCs.

### Transient upregulation of Oct4 and Nanog during early differentiation in immortalized hMSCs

To overcome loss of stem-like properties and spontaneous differentiation those hinder the expansion and application of hMSCs, we first overexpressed primary cell cultures of hMSCs with HPV 16 E6E7 and developed the KP cells [6], which were then overexpressed with hTERT. Several single-cell derived clones were isolated and 3A6, 1C5 and 3G11 were used for further analysis. All of these clones grown in monolayer in DMEM-LG supplemented with 10% FBS had a remarkably shorter population doubling time (1.9 days) compared with the parental KP cells (3.0 days). RT-PCR revealed the expression of hTERT in all these three clones. Flow cytometry also demonstrated these cells have a normal surface protein profile like the normal hMSCs (Additional file 2).

**Figure 2:**
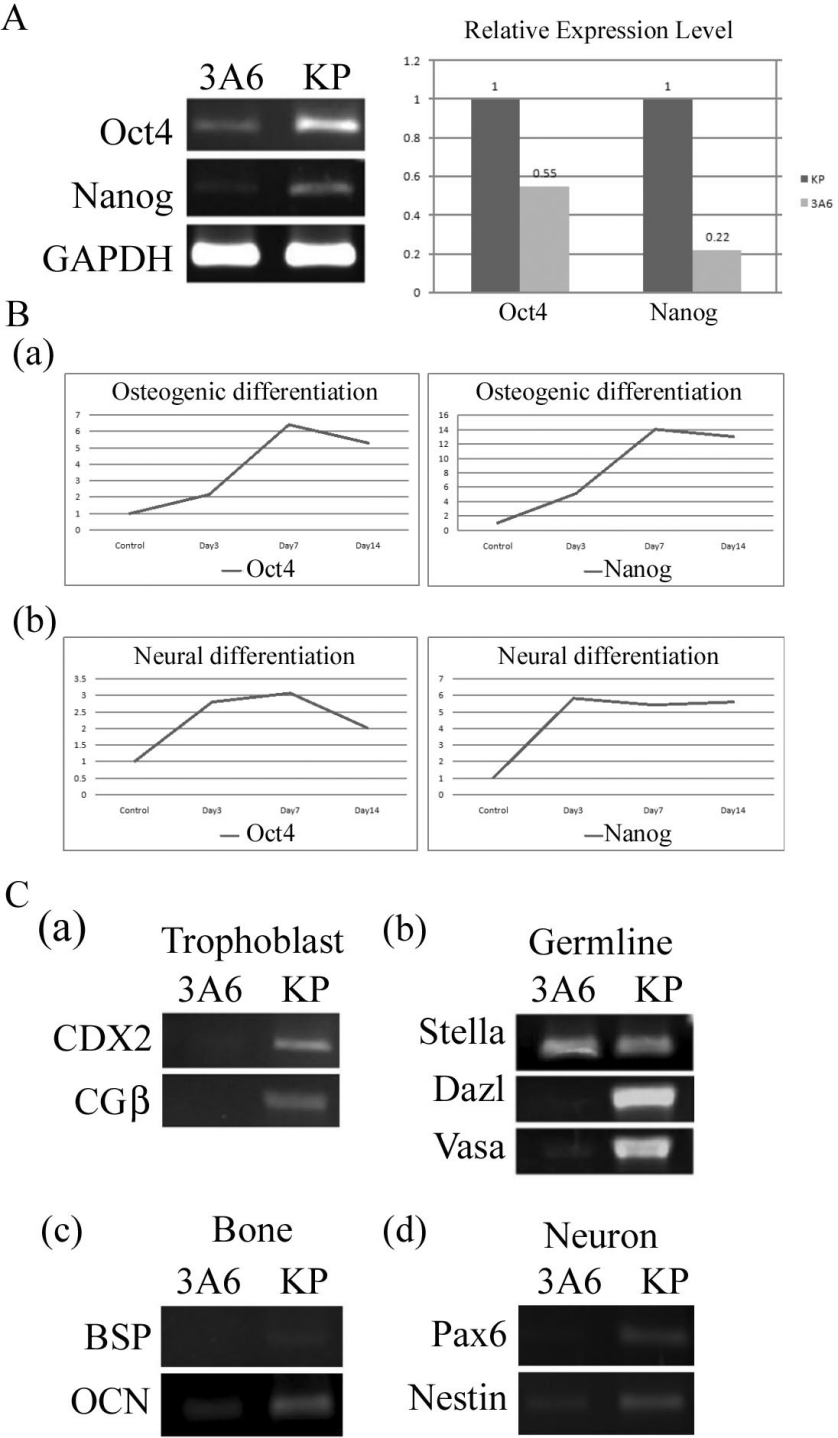
**Differential gene expression between 3A6 and KP, and alteration of pluripotency related markers during 3A6 differentiation**. **(A) **RT-PCR (left panel) and Real-time RT-PCR (right panel) analysis of pluripotency related genes in 3A6 and KP. **(B) **Differential expression of Oct4 and Nanog during (a) osteogenic and (b) neural differentiation in 3A6. **c. **RT-PCR analysis of (a) trophoectoderm (b) germline (c) osteoblastic and (d) neural lineage specific genes.

To examine if these cells increases in stem-like properties, we chose 3A6 for further evaluation. We first compared the expression levels of Oct4 and Nanog between KP and 3A6. Unexpectedly, RT-PCR and real-time RT-PCR unraveled the downregulation of both Oct4 and Nanog in 3A6 compared with KP (Figure 2A). Downregulation of the embryonic transcription factors such as Oct4 and Nanog is associated with differentiation of neural stem cells, hematopoietic stem cells and MSCs. However, an increase in Oct4 expression in ESCs causes differentiation into primitive endoderm [29], mesoderm [29] and early cardiac lineage [30]. Overexpression of Nanog also drives the expression of ectoderm markers [30]. The expression pattern of Oct4 and Nanog during differentiation is completely different between ESCs and adult stem cells such as MSCs, and should serve as an indicator to discriminate ESCs from MSCs [29-31]. We therefore induced 3A6 to undergo osteogenic and neural differentiation and examined the expression of Oct4 and Nanog. During osteogenic differentiation, we noticed a continuous upregulation of Oct4 and Nanog until day 7 followed by downregulation of both genes at day 14 (Figure 2Ba). Similarly, during neural differentiation, the upregulation of Oct4 and Nanog was observed during early differentiation (Figure 2Bb). These results indicated 3A6 has a differential gene expression of embryonic markers similar to the early differentiation of ESCs.

### Downregulation of developmental markers and lineage-specific genes in immortalized hMSCs

To clarify the blocking of spontaneous differentiation in 3A6, we compared the expression of developmental markers and lineage-specific genes between 3A6 and KP by performing RT-PCR for trophoectoderm (CDX2 and CGβ), germline (Dazl, Vasa and Scp3), osteogenic (BSP, Bone Sialoprotein and OCN, Osteocalcin) and neural (Pax6 and Nestin) specific markers. We noted a general downregulation of expression for all these genes at 3A6 compared with KP (Figure 2C), indicating 3A6 maintained in an undifferentiated state.

### Improvement of differentiation potential in immortalized hMSCs

**Figure 3:**
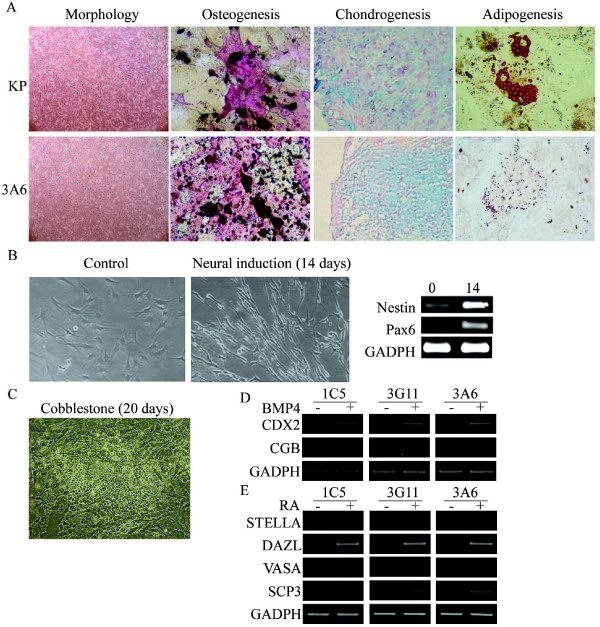
**Versatile differentiation potential of 3A6**. **(A) **Morphology without induction or with osteogenic (21 days, demonstrated by von Kossa staining), chondrogenic (21 days, demonstrated by Alcian Blue staining) or adipogenic (14 days, demonstrated by Oil Red O staining) differentiation. **(B) **Neural differentiation confirmed by the alteration of cell morphology to the round cell body with bipolar elongated cell processes, and by RT-PCR after induction with noggin for 14 days. **(C) **Cobble stone formation by co-culture with hematopoietic stem cells. **(D) **Trophoectoderm- and **(E) **germline-differentiation analyzed by RT-PCR after induction in three individual clones with BMP4 and RA, respectively.

After characterization of 3A6 and unraveling its relative quiescent state, it is of great interest if the differentiation potential of 3A6 would be sustained, enhanced and reversed to a considerably primitive state. We first examined if 3A6 sustained the normal capabilities of hMSCs, such as mesenchymal (osteogenic, adipogenic and chondrogenic) and non-mesenchymal (neural) differentiation and hematopoietic supporting potential (cobblestone forming). 3A6 had normal or elevated osteogenic and chondrogenic differentiation potential compared with one KP-derived single cell clone, whereas 3A6 had decreased adipogenic differentiation potential (Figure 3A). These data are consistent with previous studies that overexpression of hTERT increased osteogenic potential and the inverse relationship between osteogenic and adipogenic differentiation. For neural differentiation, 3A6 adopted the typical morphology of neural progenitor cells, including bipolar elongated cell processes and retracted cell bodies, and expressed neural lineage specific markers, such as Nestin and Pax6 on stimulation with noggin in serum free conditions for 14 days (Figure 3B). For co-cultured CD34+ hematopoietic stem cells with 3A6 cells, we noted the formation of cobblestone areas from hematopoietic cells that transmigrated beneath the layer of 3A6 cells (Figure 3C).

Previously, only ESCs has proven to be able to successfully differentiate toward trophoectoderm [21] and germline [28] in vitro, but Johnson and others [32] detected the expression of germline markers in bone marrow and peripheral blood, and Nayernia and others [22] further implied the germline differentiation potential of mouse MSCs. Few, if any, literature so far, however, has revealed the differentiation potential of MSCs toward trophoectoderm. To test the most versatile differentiation potential of hMSCs after ectopic expression of hTERT, we directed 3A6 and two other clones, 1C5 and 3G11 towards trophoectoderm and germline differentiation upon stimulation with BMP4 [21] and retinoic acid (RA) [33], respectively. This has been used to initiate trophoblast and germline differentiation in human ESCs. As demonstrated by RT-PCR, these cells clones started to express the trophoectoderm specific markers, such as CDX2 and CGβ (Figure 3D), and germline specific markers [28], such as Stella, Dazl, Vasa, and Scp3 (Figure 3E) after differentiation. These results together suggest these cells not only sustained normal potential as hMSCs, but also adopted the potential that was previously not belonged to hMSCs.

### Enhanced differentiation efficiency in immortalized hMSCs

**Figure 4:**
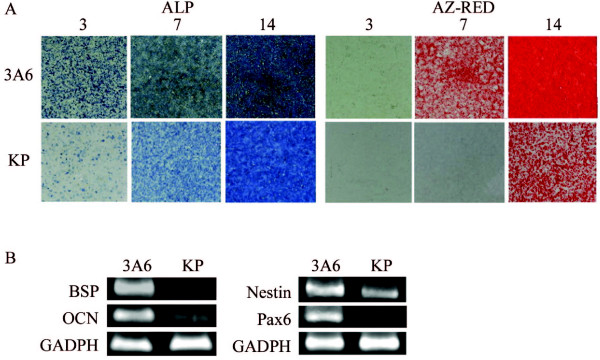
**Comparison of differentiation efficiency between 3A6 and KP**. **(A) **Histochemical staining of alkaline phosphatase (ALP) and Alizarin Red S (AZ-RED) after osteogenic induction for 3 to 14 days. **(B) **RT-PCR analysis for bone (left panel) and neuron (right panel) specific gene expression after osteogenic and neural induction for 14 days, respectively.

Besides the differentiation potential, another significant issue would be the differentiation efficiency of 3A6. Spontaneous differentiation, noted during expansion of primary hMSCs and KP, might hamper differentiation efficiency because less uncommitted cells could be directed toward specific lineage. Thus, we expected 3A6 to have better differentiation efficiency because of its less committed state. To clarify this hypothesis, we directed KP and 3A6 toward osteogenic or neural lineage and compared their differentiation efficiency by histochemical staining and lineage-specific gene expression. We observed 3A6 had higher alkaline phosphatase and Alizarin Red S staining compared with KP at day 3 to day 14 of osteogenic differentiation (Figure 4A). The expression levels of osteogenic markers-BSP and OCN were also elevated in 3A6 compared with KP during osteogenic differentiation. The expression levels of neural markers-Nestin and Pax6 were also elevated in 3A6 during neural differentiation (Figure 4B).

### Global hypomethylation of development and differentiation associated genes in immortalized hMSCs

To prove the recovery of stem-like properties after immortalization might be attributed to epigenetic remodeling, we conducted a genome-wide analysis of DNA methylation between 3A6 and KP cells, which contained about 240000 probes for 24000 CpG islands. The average methylation level of 3A6 (1.630 ± 9.456) was significantly lower than KP (1.762 ± 17.187) (Additional file 3). The numbers (percentages) of annotated genes detected as hypermethylated by the probes were 6703 (16.2%) and 7239 (17.6%) for 3A6 and KP, respectively. These results are consistent with the finding CpG islands are more frequently associated with housekeeping genes in an active state with hypomethylated DNA [34] and reveal KP has greater DNA methylation level than 3A6. Since global DNA demethylation occurs immediately following fertilization and ESCs are nearly devoid of methylation markers [17,35], the decrease in global CpG island methylation level in 3A6 further demonstrates its primitive state.

**Figure 5:**
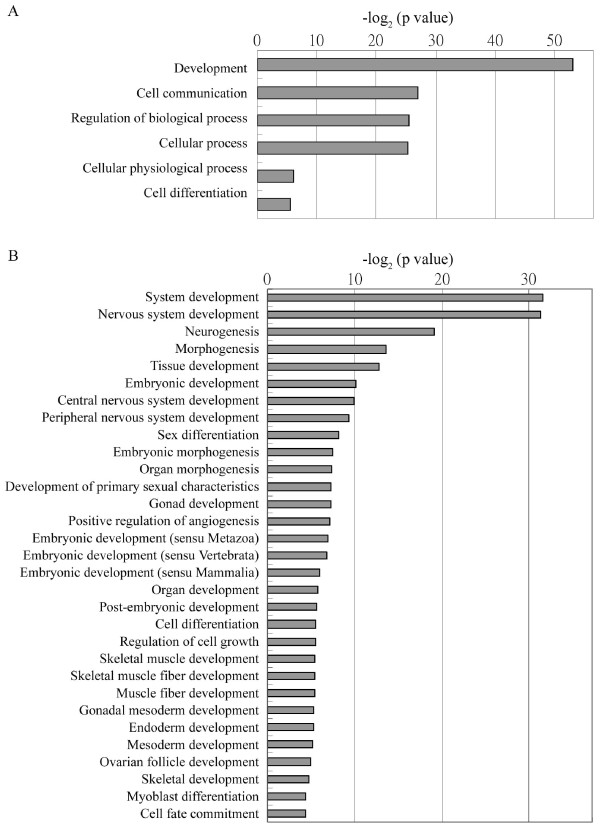
**Gene Ontology classification of genes decreased in CpG island methylation in hTERT-transfected hMSCs (A), and sub-classification of development (B)**.

Due to the decrease in numbers of hypermethylated genes in 3A6, we then analyzed genes demethylated after hTERT overexpression according to different gene categories using Gene Ontology (Figure 5). Notably, the demethylated genes were highly associated with development (p value = 1.09E-16) and cellular differentiation (p value = 0.0208). However, we didn't find a relatively higher expression level of the demethylated genes in 3A6 than in MSCs and differentiated ESCs by comparing their transcriptome microarrays (data not shown), suggesting the hypomethylated state didn't actually assure the gene expression, but rather, kept these genes in a state poised for activation.

### Decrease in expression of DNMT genes in immortalized hMSCs

Attempting to discover factors that might induce DNA demethylation in 3A6, we used real-time RT-PCR to quantify the expression level of three major DNMTs between 3A6 and KP. Surprisingly, the levels of DNMT1, DNMT3A and DNMT3B were markedly suppressed in 3A6 compared with KP (Additional file 4A). Because DNA methylation could also be controlled by the polycomb group protein, EZH2 [36], we checked the expression of EZH2 by real-time RT-PCR. The expression levels of EZH2 were not different between 3A6 and KP (Additional file 4B). In addition, ChIP-on-chip studies using anti-EZH2 antibodies revealed no correlation between demethylated genes and EZH2 binding genes in 3A6 (data not shown). From these results, the decrease in CpG island methylation in 3A6 is associated with the decrease in DNMT gene expression, but not EZH2 associated.

### The gene expression profile of immortalized hMSCs is similar with that of ESCs

**Figure 6:**
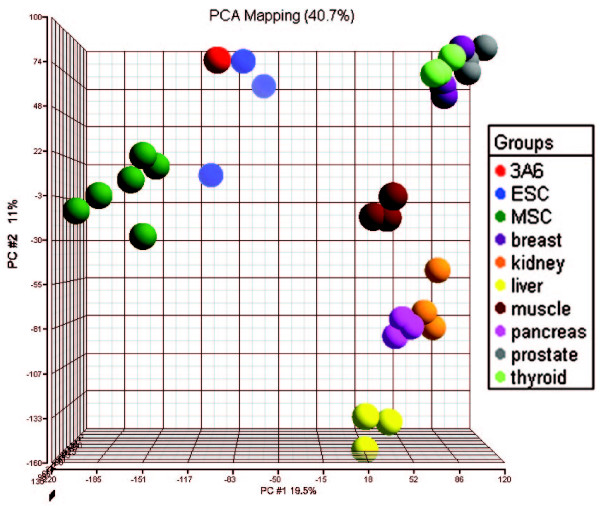
**The expression profile of 3A6 immortalized hMSCs converges toward embryonic stem cells**. Principal component analysis comparing the gene expression profiles of 3A6, embryonic stem cells (ESC), mesenchymal stem cells (MSC), and various tissues using all the transcriptome data. Each plotted data point represents a single profile.

To gain insight into the convergence of 3A6 toward ESCs, we compared the expression profile of 3A6 with various normal human tissues, MSCs and ESCs. This data set therefore contained different tissues from embryo, endoderm, epithelial, or mesenchymal origins. The expression profiles of each chip were compared using principal component analysis (PCA) to discover the similarity of the expression profiles within and across the cells or tissues. PCA using all probe sets showed ESC and MSC each formed a distinct group and were quite different from all the normal human tissues. Interestingly, the 3A6 expression profile located very close to ESCs rather than near MSCs, signaling the expression profile of 3A6 converged toward ESCs (Figure 6).

## Discussion

To circumvent the problems associated with expanded hMSCs, we found that ectopic expression of HPV 16 E6E7 and hTERT enhanced proliferation and stem-like properties, and blocked spontaneous differentiation in primary culture of hMSCs. Surprisingly, all of the three examined cell clones had differentiation potential far beyond the normal hMSCs. They expressed trophoectoderm and germline specific markers at day 7 of induced differentiation with BMP4 and RA, respectively. Besides unlimited differentiation potential, we further showed these cells displayed higher osteogenic and neural differentiation efficiency than their parental cells. The increased differentiation efficiency was attributable to the decrease in committed cells that have spontaneously undergone differentiation and might be limited in directed differentiation potential.

DNA methylation and chromatin structure are major epigenetic factors that regulate gene expression [37]. Increase in CpG island methylation was noticed during ESC differentiation [38,39] and deleting the three major DNMTs would cause hypomethylation and thorough blockage of differentiation of ESCs [40,41]. These findings plus the fact global methylation marks are erased after fertilization and formation of embryo, and increase during in vitro expansion [42] suggest the CpG island methylation profile may serve as an indicator of "primitiveness" of stem cells. Therefore, the decrease in CpG island methylation in 3A6 suggests its increase in primitiveness. More importantly, DNA demethylation occurred mainly in the CpG islands of development and differentiation associated genes, and ensured these genes the accessibility for activation upon cues of stimulation and further explained the unlimited differentiation potential. To elucidate if the enhancement of stem-like properties and blockage of spontaneous differentiation by hTERT overexpression is restricted merely to the immortalized cell line, we also inspected the effects of ectopic expression of hTERT in primary hMSCs. Although overexpression of hTERT inhibited the expressions of DNMTs (Additional file 5), it did not induce a significant change in pluripotency and lineage gene expression. These results suggest hTERT alone or downregulation of DNMTs is not enough to trigger reversion of stem-like properties in hMSCs, which needs a combinational activation of many factors or molecules as demonstrated previously [43].

In the current study, CpG island hypomethylation did not induce an increase in the average gene expression level in 3A6. Weber [15] clarified most of the unmethylated promoters with high CpG frequency (HCPs) remain inactive. Mikkelsen and others [44] further explored the chromatin state of HCPs in ESCs and revealed monovalent promoters (H3K4me3) generally regulate genes with "housekeeping" functions, and otherwise, bivalent promoters (H3K4me3 and H3K27 me3) are associated with genes related to key developmental transcription factors. Most importantly, they found low activity of bivalent HCPs, compatible with the findings that most of the development associated genes are quiescent in pluripotent cells. Therefore, the low activity of demethylated development-associated genes in 3A6 might be due to transient repression by chromatin modifications, and indeed the hypomethylated state of these genes enable them to recapitulate expression upon later development or cellular differentiation.

Despite further investigations needed to elucidate exact demethylation mechanism, the effect of global DNA demethylation on stem-like properties and behavior of stem cells is still of great significance. Taylor and others first described that treatment with 5-AzaC increased the differentiation potential of C3H/10T1/2 cells [19]. Similarly, 5-AzaC also induced dedifferentiation in partially differentiated ESCs [18] or trophoblast stem cells [20]. These findings support our findings that 3A6 with a significant global decrease in CpG island methylation level behaved like ESCs and such alteration in stem-like properties might be achieved by DNA demethylation of development and differentiation associated genes after immortalization.

Therefore, transfection of hMSCs with HPV 16 E6E7 and hTERT might elicit change of epigenetic marks to reverse stem-like properties, which finally contributes to unlimited differentiation potential and increased differentiation efficiency. However, there are several limitations to this study. First, because the cells require transfection to improve stem cell properties and prevent spontaneous differentiation, these transfected cells may not be applied for clinical uses. Second, the increase of multipotency to trophoectoderm and germ cells was only demonstrated by the expression of several lineage markers, without any in vivo study it is still limited to define these potential. Although the hidden ulterior connection between overexpression of these genes and epigenetic remodeling in stem cell biology is needed, the current results are a great step forward in establishing the feasibility and applicability of adult stem cells in future clinical applications.

## Competing interests

The authors declare that they have no competing interests.

## Authors' contributions

CCT performed research and analyzed data, CLC designed research, performed research and wrote the paper, HCL contributed vital new reagents or analytical tools, YTL performed research, HWW contributed vital new reagents or analytical tools and analyzed data, LTH contributed vital new reagents or analytical tools and wrote the paper, SCH designed research, contributed vital new reagents or analytical tools and wrote the paper. All authors read and approved the final manuscript.

## Supplementary Material

Additional file 1 **Primer sets and NCBI reference sequence number**. Click here for file

Additional file 2 **(A) Detection of hTERT mRNA expression in 1C5, 3G11 and 3A6, and (B) Characterization of CD molecules in 3A6**. Cytofluorimetric profiles of 3A6 reacted first with (solid line) or without (broken line) mouse MAbs specific for each marker, and second with fluorescein-labeld antimouse Ig antibody. Click here for file

Additional file 3 **Box plots show average methylation levels of genes contain CpG islands**. P value was calculated using a *t*-test. Click here for file

Additional file 4 Real-time RT-PCR analysis of expression levels of (A) DNMT1, DNMT3A and DNMT3B, and (B) EZH2 in 3A6 and KP. Click here for file

Additional file 5 **(A) RT-PCR analysis of expression levels of hTERT and GAPDH, and (B) Real-time RT-PCR analysis of expression levels of DNMT1, DNMT3A and DNMT3B in primary human mesenchymal stem cells transfected with plasmids carrying control and hTERT vectors**. Data are presented as mean ± S.D. *p < 0.01 compared with control as calculated using a *t*-test. Click here for file
