# Supplementary material for: Overexpression of hTERT increases stem-like properties and decreases spontaneous differentiation in human mesenchymal stem cell lines
Source: J Biomed Sci. 2010 Jul 29;17(1):64. doi: 10.1186/1423-0127-17-64 (PMC2923118; doi:10.1186/1423-0127-17-64)
Supplement: Additional file 1 — Primer sets and NCBI reference sequence number. [file 1423-0127-17-64-S1.PDF]

Supplementary Table 1. Primer sets and NCBI reference sequence number

Name	Primer sequence	Size	NCBI reference sequence number
**GAPDH**	F: GTTCCAATATGATTCCACCC<br>R: TGAGTCCTTCCACGATACC	400	M33197.1
**h-TERT**	F: AGCCAGTCTCACCTTCAACCGC<br>R: GGAGTAGCAGAGGGAGGCCG	272	NM_198255.2
**Oct4**	F: CGACCATCTGCCGCTTTGAG<br>R: CCCCCTGTCCCCCATTCCTA	543	NM_203289.4
**Nanog**	F: TGCCTCACACGGAGACTG<br>R: GCTATTCTTCGGCCAGTT	353	NM_024865.2
**Runx2**	F : GTTTGTTCTCTGACCGCCTC<br>R : CCAGTTCTGAGGCACCTGAAA	317	NM_001024630.3
**BSP**	F : TCAGCATTTTGGGAATGGCC<br>R : GAGGTTGTTGTCTTCGAGGT	657	NM_004967.3
**OCN**	F : CATGAGAGCCCTCACA<br>R : AGAGCGACACCCTAGAC	310	NM_199173.3
**Nestin**	F: CAGCTGGCGCACCTCAAGATG<br>R: AGGGAAGTTGGGCTCAGGACTGG	208	NM_006617.1
**Pax6**	F: AACAGACACAGCCCTCACAAACA<br>R: CGGGAACTTGAACTGGAACTGAC	274	NM_001604.4
**Sox17**	F: CGCACGGAATTTGAACAGTA<br>R: GGATCAGGGACCTGTCACAC	181	NM_022454.3
**FoxA2**	F: AAGTGGGGGTCGAGACTTTG<br>R: CTGCAACAACAGCAATGGAG	299	NM_021784.4
**cTn-I**	F: AATTGCAGCTGAAGACTCTG<br>R: GACTTTTGCCTCTATGTCGT	218	NM_000363.4
**Nkx 2.5**	F: GAGAGTTTGTGGCGGCGATT<br>R: CGACGGCGAGATAGCAAAGG	205	NM_004387.2
**Sox2**	**F:** ATGCACCGCTACGACGTGA<br>**R:** CTTTTGCACCCCTCCCATTTT	437	NM_003106.2
**Stella**	F: GTTACTGGGCGGAGTTCGTA<br>R: TGAAGTGGCTTGGTGTCTTG	174	XR_017793.3
**Dazl**	F: AGCCACGTCCTTTGATTTT<br>R: TAAGCACTGCCCGACTTCTT	335	NM_001351.2
**Vasa**	F: AAGAGAGGCTATCGAGATGGA<br>R: CGTTCACTTCCACTGCCACTTCTG	238	NM_024415.2
**Scp3**	F: GAGCCTATGACTTTGAGACTG<br>R: TCTAAATCCCACTGCTGAAAC	349	NM_153694.3
**CDX2**	F: GAACCTGTGCGAGTGGATGCG<br>R: GGTCTATGGCTGTGGGTGGGAG	563	NM_001265.3
**CGß**	F: CAACACCACCATCTGTGC<br>R: CTTTATTGTGGGAGGATC	354	NM_000737.2
**Gata4**	F: CTGGCCTGTCATCTCACTACG<br>R: GGTCCGTGCAGGAATTTGAGG	262	NM_002052.3
**Gata6**	F: TTCTAACTCAGATGATTGCAGC<br>R: GCTGCACAAAAGCAGACACG	299	NM_005257.3
**DNMT1**	F: ACCGCTTCTACTTCCTCGAGGCCTA<br>R: GTTGCAGTCCTCTGTGAACACTGTGG	335	NM_001379.2
**DNMT3A**	F: CACACAGAAGCATATCCAGGAGTG<br>R: AGTGGACTGGGAAACCAAATACCC	551	NM_175629.1
**DNMT3B**	F: AATGTGAATCCAGCCAGGAAAGGC<br>R: ACTGGATTACACTCCAGGAACCGT	190	NM_175849.1
**EZH2**	F: GTGGAGAGATTATTTCTCAAGATG<br>R: CCGACATACTTCAGGGCATCAGCC	289	NM_152998.1
