# Supplementary figures and images for: Overexpression of hTERT increases stem-like properties and decreases spontaneous differentiation in human mesenchymal stem cell lines
Source: J Biomed Sci. 2010 Jul 29;17(1):64. doi: 10.1186/1423-0127-17-64 (PMC2923118; doi:10.1186/1423-0127-17-64)

A

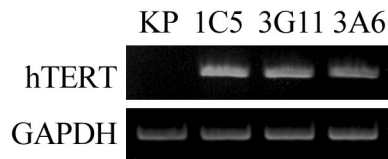

B

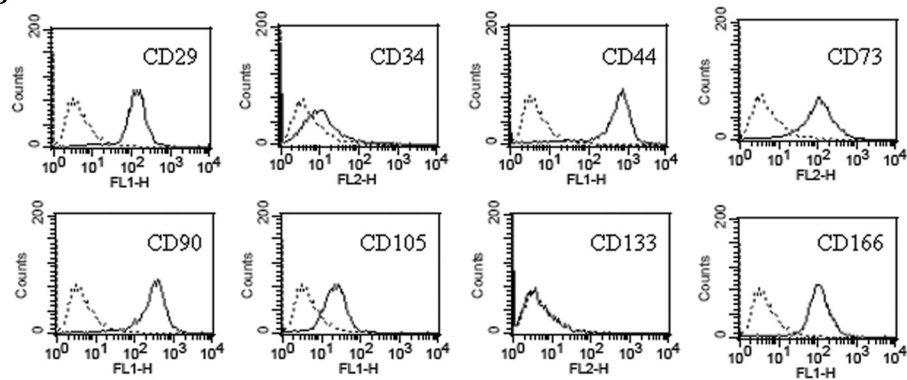

Supplementary Fig. 1

Supplement: Additional file 2 — (A) Detection of hTERT mRNA expression in 1C5, 3G11 and 3A6, and (B) Characterization of CD molecules in 3A6. Cytofluorimetric profiles of 3A6 reacted first with (solid line) or without (broken line) mouse MAbs specific for each marker, and second with fluorescein-labeld antimouse Ig antibody. [file 1423-0127-17-64-S2.PDF]

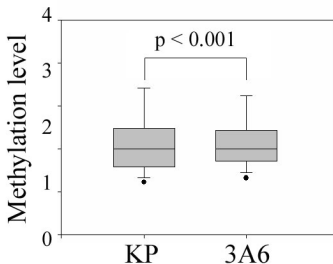

Supplementary Fig. 2

Supplement: Additional file 3 — Box plots show average methylation levels of genes contain CpG islands. P value was calculated using a t-test. [file 1423-0127-17-64-S3.PDF]

A

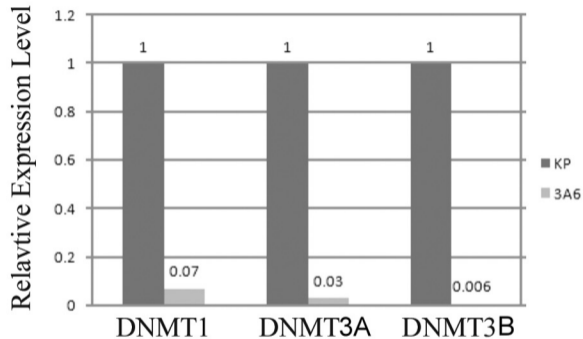

B

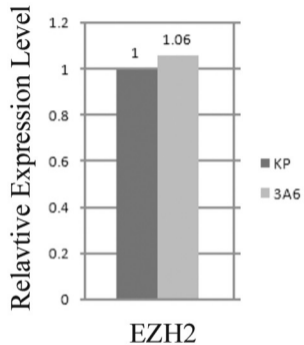

Supplementary Fig. 3

Supplement: Additional file 4 — Real-time RT-PCR analysis of expression levels of (A) DNMT1, DNMT3A and DNMT3B, and (B) EZH2 in 3A6 and KP. [file 1423-0127-17-64-S4.PDF]

A

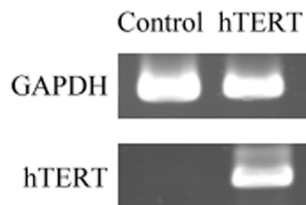

B

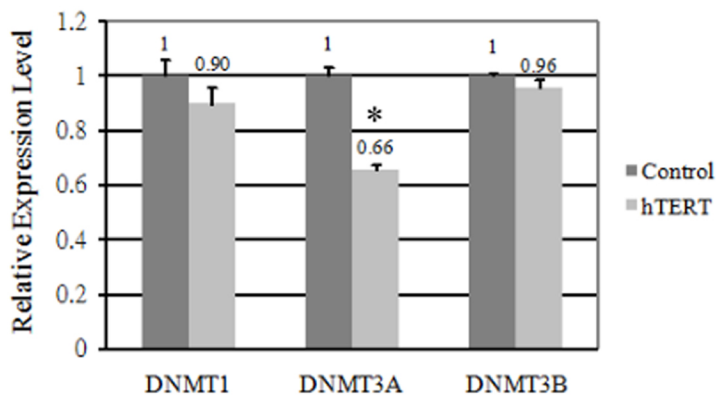

Supplementary Fig. 4

Supplement: Additional file 5 — (A) RT-PCR analysis of expression levels of hTERT and GAPDH, and (B) Real-time RT-PCR analysis of expression levels of DNMT1, DNMT3A and DNMT3B in primary human mesenchymal stem cells transfected with plasmids carrying control and hTERT vectors. Data are presented as mean ± S.D. *p < 0.01 compared with control as calculated using a t-test. [file 1423-0127-17-64-S5.PDF]
